# Niacin Alternatives for Dyslipidemia: Fool’s Gold or Gold Mine? Part I: Alternative Niacin Regimens

**DOI:** 10.1007/s11883-016-0563-8

**Published:** 2016-02-15

**Authors:** Richard L. Dunbar, Harsh Goel

**Affiliations:** Department of Medicine, Division of Cardiovascular Medicine, Perelman School of Medicine at the University of Pennsylvania, Philadelphia, PA USA; Division of Translational Medicine and Human Genetics, Perelman School of Medicine at the University of Pennsylvania, 3600 Spruce Street, 9-010 Maloney Building, Philadelphia, PA 19104 USA; Institute for Translational Medicine and Therapeutics, Perelman School of Medicine at the University of Pennsylvania, Philadelphia, PA USA; The Cardiovascular Institute, Perelman School of Medicine at the University of Pennsylvania, Philadelphia, PA USA; Institute for Diabetes, Obesity, and Metabolism, Perelman School of Medicine at the University of Pennsylvania, Philadelphia, PA USA; Department of Medicine, York Hospital, 1001 S. George Street, York, PA 17403 USA

**Keywords:** Niacin, Nicotinic acid, Hyperlipidemia, Niacin conjugates, Niacin prodrugs, Lipids

## Abstract

Niacin was the first drug demonstrating lowered cholesterol prevents coronary heart disease (CHD) events, with two clinical CHD outcome studies establishing a cardioprotective niacin regimen: 1 g thrice daily with meals. Though cardioprotective, skin toxicity limits niacin’s use, fostering several variations to improve tolerability. One of these, an extended-release (ER) alternative, proved immensely successful commercially, dominating clinical practice despite departing from the established regimen in several critical ways. Hence, improved tolerability may have come at the cost of diminished efficacy, posing a conundrum: Does it still help the population at risk for CHD to broaden a drug’s acceptance by “watering it down”? This question is crucial at this stage now that the ER alternative failed to recapitulate the benefits of the established cardioprotective niacin regimen in two trials of the alternative approach: AIM-HIGH and HPS2-THRIVE. Part I of this review discusses how vastly the ER alternative departs from the established cardioprotective regimen, why that is important physiologically, and how it may explain the findings of AIM-HIGH and HPS2-THRIVE. Given important gaps left by statin therapy, the established cardioprotective niacin regimen remains an important evidence-based therapy for the statin intolerant or statin averse.

**Electronic supplementary material:**

The online version of this article (doi:10.1007/s11883-016-0563-8) contains supplementary material, which is available to authorized users.

## Introduction

Almost 60 years ago, Altschul discovered NIcotinic ACid vitamIN (NI’AC’IN) suppressed lipids in man [1]. Subsequently found to lower cholesterol and triglycerides (TGs) among the atherogenic lipoproteins (non-HDL-C and LDL-C) and raise HDL-C [2, 3], niacin became the first to demonstrate that lowering cholesterol prevents myocardial infarction (MI) [3] and, eventually, all-cause mortality [4]. Thus, niacin became foundational to treating hypercholesterolemia to prevent MI. Cardioprotection was shown with 1 g thrice daily with meals in two distinct niacin formulations: (1) immediate-release (IR) niacin [3] and, later, (2) a longer-releasing niacin pro-drug, pentaerythrityl tetranicotinate [5]. Accordingly, several consensus groups endorsed niacin to prevent hard coronary heart disease (CHD) events: non-fatal MI and cardiac death [6–9]. Attempts to capitalize on the benefits of this regimen included titration well beyond 3 g/day to reach specific lipid targets [10–13], using up to 12 g/day [10]. Another strategy was to re-tool the pharmacokinetics, radically altering the dosing schedule to enhance tolerability, the most commercially successful being prolonged-release alternatives. An extended-release (ER) alternative attracted major backing as Niaspan, but counter-intuitively, at a maximum dose restricted to only 2 g/day. It is critical to appreciate the ER alternative is an alternative rather than an equivalent to the established cardioprotective regimen, the former bearing the burden of proof in terms of safety, lipid efficacy, and, ultimately, CHD event reduction.

## The Alternative Versus the Established Niacin Regimen

Though fairly safe, the ER alternative is at a major disadvantage compared to other niacin formulations, in that the FDA limited the maximum dose to 2 g/day, whereas the established cardioprotective dose is 3 g/day, raising the obvious question: For practical purposes, is 2 g really less than 3 g, or is it “close enough for government work?” Ultimately, this would require a new round of outcome trials. Importantly, IR-niacin continues to improve lipids above 2 g/day, as demonstrated by Knopp et al. in a head-to-head trial comparing IR-niacin to the ER alternative among hyperlipidemics [14]. At the low end, both formulations lowered lipids comparably at only half the usual dose (i.e., 1.5 g daily). However, when titrated to the established cardioprotective dose (i.e., 3 g daily), IR-niacin clearly exceeded the ER alternative’s capability to lower lipids [14]. Considering the FDA approved IR-niacin up to 6 g/day, the top dose of the ER alternative is only 2/3 the established cardioprotective dose and only 1/3 the maximum approved dose of IR-niacin, raising grave doubts as to its equivalency to the established cardioprotective regimen.

Unfortunately, the problem for the ER alternative runs much deeper than profound underdosing. Departing further, the ER alternative was dosed entirely at bedtime rather than throughout fed portion of the day, abandoning the successful approach of both trials establishing CHD benefit. Nevertheless, the ER alternative was touted as a gentler alternative to IR-niacin, and absent contrary evidence presumed cardioprotective long enough to dominate clinical use, encouraged by small studies demonstrating atheroprotection [15–17].

**Fig. 1:**
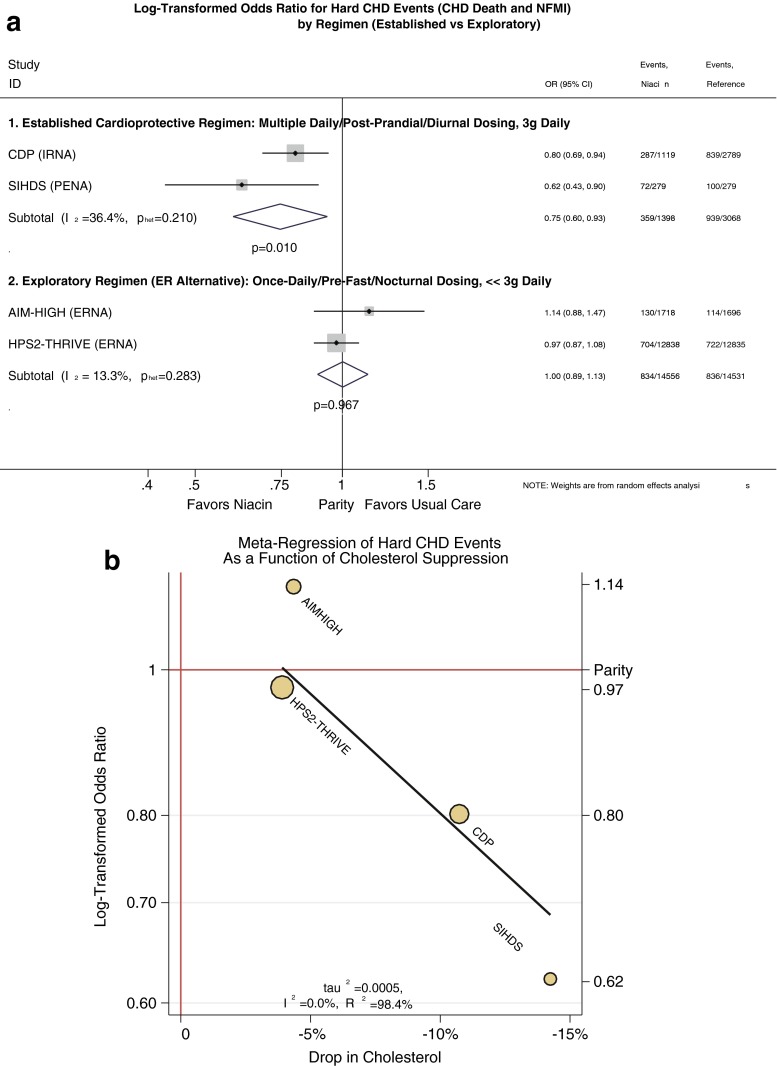
**a** Meta-analysis of odds ratio for hard CHD events (CHD death and nonfatal myocardial infarction) comparing the exploratory ER alternative to the established cardioprotective regimen. The event rates for active and comparator groups are as reported in the four cardiovascular outcomes trials (CDP, SIHDS, AIM-HIGH, and HPS2-THRIVE) [3, 5, 18••, 19••]. We used random-effects meta-analysis techniques to pool the corresponding log-transformed odds ratios (OR) by the metan procedure in Stata v14. Initially, we analyzed the exploratory regimen as if it were equivalent to the established cardioprotective regimen, pooling all four trials. Unsurprisingly, treating such different approaches as equivalents revealed a high degree of heterogeneity, *I* ^2^, as high as 73 % (*p* = 0.012), indicating the overwhelming amount of variation between trials is attributable to heterogeneity. Thus, pooling the trials proved unsupportable. In marked contrast, treating the exploratory regimen as distinct from the established cardioprotective regimen rendered heterogeneity insignificant and inconsequential, as shown. Among trials of the exploratory regimen, *I* ^2^ was very low (13 %, *p* = 0.3), indicating agreement. Specifically, these trials agreed on inefficacy of the alternative regimen (OR = 1.0, *p* = 1). Likewise, among trials of the established cardioprotective regimen, *I* ^2^ was low (36 %, *p* = 0.2), again indicating agreement. Specifically, these trials agreed on efficacy of the established cardioprotective regimen (OR = 0.75, *p* = 0.01). The high heterogeneity when pooling all four trials but low heterogeneity when distinguishing the exploratory from the established regimen further supports the concept that the alternative is not an equivalent with respect to outcomes. This is even more apparent when considering the clinical effects on hard CHD events (i.e., no benefit from the alternative, but an odds ratio of 0.75 for the established regimen). Clinically, this analysis affirms a role for the established regimen and denies a role for the ER alternative. *CI* confidence interval, *IRNA* immediate-release niacin, *PENA* pentaerythrityl tetranicotinate, *ERNA* extended-release niacin. **b** Meta-regression between log-transformed odds ratio for hard CHD events and percent change in cholesterol. As with Fig. 1, we assessed the odds ratios for hard CHD events for the four cardiovascular outcome trials, now evaluating whether cardioprotection is consistent with the cholesterol hypothesis. Specifically, we regressed the odds ratio for CHD against the drop in total cholesterol for each study by the metareg procedure in Stata v 14. Meta-regression revealed an extraordinarily strong relationship, with virtually no heterogeneity (*I* ^2^ = 0 %) and nearly perfect correlation (*R* ^2^ = 98 %). At the low end, it is intuitive that the alternative regimen of AIM-HIGH and HPS2-THRIVE conferred no meaningful CHD benefit, as the reduction in cholesterol was modest at best (∼5 %). This accords with the cholesterol hypothesis, which predicts that minimal reductions in cholesterol translate to minimal benefits on CHD events. On the other hand, the regimens of CDP and SIHDS present progressive reductions in cholesterol that correspond to dose-responsive CHD benefits (dose referring to cholesterol reduction). This also accords with the cholesterol hypothesis, which predicts that greater reductions in cholesterol should translate to greater CHD benefits in a dose-responsive fashion. In addition to distinguishing the alternative regimen from the established regimen, the strong linear relationship between cholesterol lowering and CHD prevention by niacin supports the concept that the lipid-targeting strategy would improve on the already-substantial benefits of the established regimen

Commendably, major stakeholders of the exploratory regimen mounted vascular event outcome trials to prove that the ER alternative could limit cardiovascular events as IR-niacin and pentaerythrityl tetranicotinate had done, this time against a statin background. The aborted AIM-HIGH trial [18••] was almost as large as the Coronary Drug Project (CDP), if much shorter [3], and the completed HPS2-THRIVE trial was even larger [19••], together promising a robust test of the alternative regimen. Surprisingly to many, the ER alternative failed to recapitulate benefits of the established cardioprotective regimen. Perhaps the most obvious explanation for their apparent null results is the exploratory ER regimen compromised several critical features of the established cardioprotective regimen (Table 1). Tellingly, in outcome trials, 1 g thrice daily with meals reduces hard CHD events singly or pooled, with a pooled odds ratio (OR) of 0.75 (95 % confidence interval [CI] = 0.60 to 0.93, *p* = 0.01, Fig. 1a). In contrast, such benefits appear well beyond the reach of the exploratory regimen of 2-g single dose before the overnight fast (OR = 1.00, 95 % CI = 0.89 to 1.13, *p* = 0.97).

**Table 1:** Major differences between the established cardioprotective regimen and the ER alternative regimen

Strategy employed by clinical event trials	Established cardioprotective regimen		Exploratory regimen (cardioprotection indeterminate)
Dose tested	3 g/day	>>	2 g/day
Exposures per day	3	>>	1
Portion of day exposed	Diurnal	≠	Nocturnal
Fed/fasting state during drug exposure	Postprandial (breakfast, lunch, and supper)	≠	Post-absorptive (overnight fast)
Adjusted lipid changes^a^			
TC	−12.6 %	>	−7.4 %
TG	−26.4 %	>	−19.5 %
LDL	−15.4 %	>	−9.5 %
HDL	+22.5 %	≈	+21.9 %
Pharmacokinetics of formulation	Immediate-release niacin,	<	Extended-release niacin
	longer-release pro-drug	≈	

^a^Lipid changes are from a meta-analysis by Birjmohun et al. (J Am Coll Cardiol 2005;45:185–97)

Whether the exploratory alternative regimen is actually equivalent is crucial, because it affects how the established cardioprotective regimen might be used. Substantially underdosing the alternative is intuitively cause for pause; indeed, there is much evidence supporting the dose–response relationship between cholesterol suppression and CHD risk (Fig. 1b) [20, 21]. There is also evidence that nocturnally dosing the ER alternative does not lower postprandial lipids [22•, 23], thus, failing to suppress an important pool of atherogenic lipids [24, 25]. We found meal-time dosing reduced postprandial lipidemia [22•], which itself may be atheroprotective [25, 26]. In contrast, nocturnal dosing of the ER alternative confers no such benefit [23]. The AIM-HIGH authors also implicate nocturnal dosing because niacin causes a multi-fold rebound of free fatty acids (FFA) several hours post-dose. They contend “this metabolic perturbation repeated every night could promote CV events via impaired myocardial fuel supply, subsequent excess in fatty acid anion concentrations, and/or a counter-regulatory hormone response, including catecholamines.” [27]. Conversely, they reason that the cardioprotective meal-time regimen might forego these ill effects. At this point, to continue to presume that the alternative regimen is equivalent to the established cardioprotective regimen strains credibility.

## Revisiting the Established Cardioprotective Regimen

Clearly, a convincing failure of an alternative to live up to the standard invalidates the alternative; however, such failure does not invalidate the standard. Now that the presumption of equivalency between the two opposing regimens has been debunked, we think it is important to re-visit the evidence supporting the established cardioprotective regimen. The CDP compared five cholesterol-lowering strategies to placebo among MI survivors [28]. At 1 g thrice daily, IR-niacin prevented recurrent MI after 5 years, whereas the other four strategies failed [3]. Interestingly, among the four failed therapies was a fibrate; thus, in a head-to-head matchup, niacin proved cardioprotective whereas a fibrate failed [3]. A second study affirmed the pro-drug pentaerythrityl tetranicotinate 1 g thrice daily combined with the same failed fibrate from the CDP, preventing MI after 5 years in the Stockholm Ischemic Heart Disease Study (SIHDS) [5]. Since the fibrate had proven powerless to prevent MI as monotherapy, reconciling the results with CDP further commends niacin as a cardioprotective agent. Because it used monotherapy, CDP is by far the cleaner study and more definitively establishes niacin 1 g thrice daily as cardioprotective. Having used combination therapy, the SIHDS is corroboratory but less definitive than the CDP. Furthermore, having used a longer acting pro-drug [29], we think SIHDS supports the concept that dosing 1 g thrice daily is more important than the release rate of free nicotinic acid. Long-term follow-up of CDP revealed the niacin-treated group had lower all-cause mortality despite stopping niacin after 5 years [4]. Such legacy benefits imply prior niacin use fundamentally altered the long-term disease course rather than simply keeping it in abeyance during active treatment, the sine qua non for disease-modifying therapy. Long the only lipid-lowering therapy to decisively prevent CHD events, niacin became first-line therapy along with bile acid sequestrants, as reflected by the Adult Treatment Panel (ATP) guidelines from 1988 to 2001 [30, 31]. Per public records, between 1957 and 2015, the US FDA approved 39 niacin drug/dose combinations as prescription-only pharmaceuticals to improve lipids. These include 26 approved applications featuring niacin: 16 for IR niacin and, more recently, 10 for the ER alternative [32•]. Importantly, the FDA approved the broad claim that “In patients with a history of myocardial infarction and hyperlipidemia, niacin is indicated to reduce the risk of recurrent nonfatal myocardial infarction.” (http://www.rxabbvie.com/pdf/niaspan.pdf).

## The Major Problem with Niacin

Despite benefits, a major limitation of niacin has been the universally disagreeable dermal side effect, dubbed “flushing”, a misleading metonym because it emphasizes one of the least bothersome symptoms, skin reddening [33]. Though flushing or *rubor* is cosmetically unappealing, the bothersome symptoms arise from dermal *calor*, *dolor*, *tumor*, and especially, pruritus. Since “flushing” misdirects attention from more bothersome symptoms, we prefer the more general term niacin-associated skin toxicity (NASTy). Despite demonstrable CHD benefits, niacin fell short of its potential due to NASTy effects. Over the years, several measures emerged to improve tolerability: slow titration, meal-time dosing, pretreatment with non-steroidal anti-inflammatory drugs (NSAIDs), especially aspirin, and developing prolonged-release alternatives dosed once at bedtime [14, 34–36]. Despite partially improving symptoms, overall niacin adoption remained poor.

### Statins Displaced Niacin…

High incidence and morbidity/mortality of CHD and the strong association with dyslipidemia, along with improved outcomes from cholesterol suppression spawned new lipid-lowering drugs, most successfully the statins. Picking up where niacin left off, statins multiply proved the cholesterol hypothesis, refined as the LDL hypothesis. More effective at lowering LDL-C [37, 38], better tolerated, and enjoying superlative evidence for preventing CHD events [39], statins displaced niacin as first-line therapy, reflected in the 2001 ATP and subsequent guidelines [9, 40, 41].

### …but Statins Leave Much to Be Desired

Despite strengths, statins have significant limitations: Though better tolerated than niacin, a very conservative estimate is that 10 % of patients remains statin intolerant [42, 43]. Additionally, statin response varies substantially, with up to 40 % of patients unable to achieve lipid goals with monotherapy, thus leaving a big therapeutic gap in at-risk populations [44•]. Moreover, despite reducing hard CHD events 25–35 % among those who will take them, statins leave substantial residual risk [45]. Hence, there is a dire need for both an alternative and adjunct to statins. Obviously, besides exploiting novel therapeutic pathways, one logical and less costly approach should be to study the cumulative effect of established cardioprotective agents added to statins. Supporting this, when added to baseline statin therapy, niacin further drops LDL-C and triglycerides and raises HDL-C, regressing atherosclerotic plaque [15, 17, 46] providing a sound basis to study clinical outcomes with the established cardioprotective regimen against a statin background. Unfortunately, this has never been investigated, and lamentably, failure of the exploratory ER alternative could frustrate attempts to fund such a test of the established cardioprotective regimen.

### The Spectacular Failure of the Extended-Release Alternative

The two failed trials of the exploratory regimen had very different purposes. Most straightforward, the HPS2-THRIVE trial assessed outcomes by adding the exploratory ER alternative regimen to the background statin therapy [19••]. The AIM-HIGH trial was designed for a much different reason: to test the “HDL hypothesis”, namely that raising HDL-C per se would decrease the residual risk beyond the reach of statins [47].

### The HPS2-THRIVE Trial: A Relatively Straightforward Design to Test the Exploratory Niacin Alternative

The HPS2-THRIVE enrolled patients with established cardiovascular disease but without any pre-specified lipid thresholds for eligibility. Standardization of baseline therapy with simvastatin 40 mg/day ± ezetimibe 10 mg/day was followed by randomization to ER niacin (ERN)-laropiprant 2 g/40 mg daily or placebo. On baseline statin therapy, ERN-laropiprant lowered LDL-C further, −10 mg/dL (−16 %), and raised HDL-C, +6 mg/dL (+14 %), compared to placebo over a median follow-up of 4 years. There was no difference between groups in any primary or secondary event end-points besides a significant 10 % reduction in revascularization procedures in the ERN-laropiprant group. Notably, however, baseline LDL-C was 63 mg/dL and HDL-C was 44 mg/dL, making the results difficult to extrapolate to the real-world population of dyslipidemics. Thus, subjects may have simply been too well treated to make any further impact. Perhaps hypercholesterolemia is analogous to other atherosclerotic risks, such as diabetes or hypertension: At some point, further treatment has diminished or no returns.

**Fig. 2:**
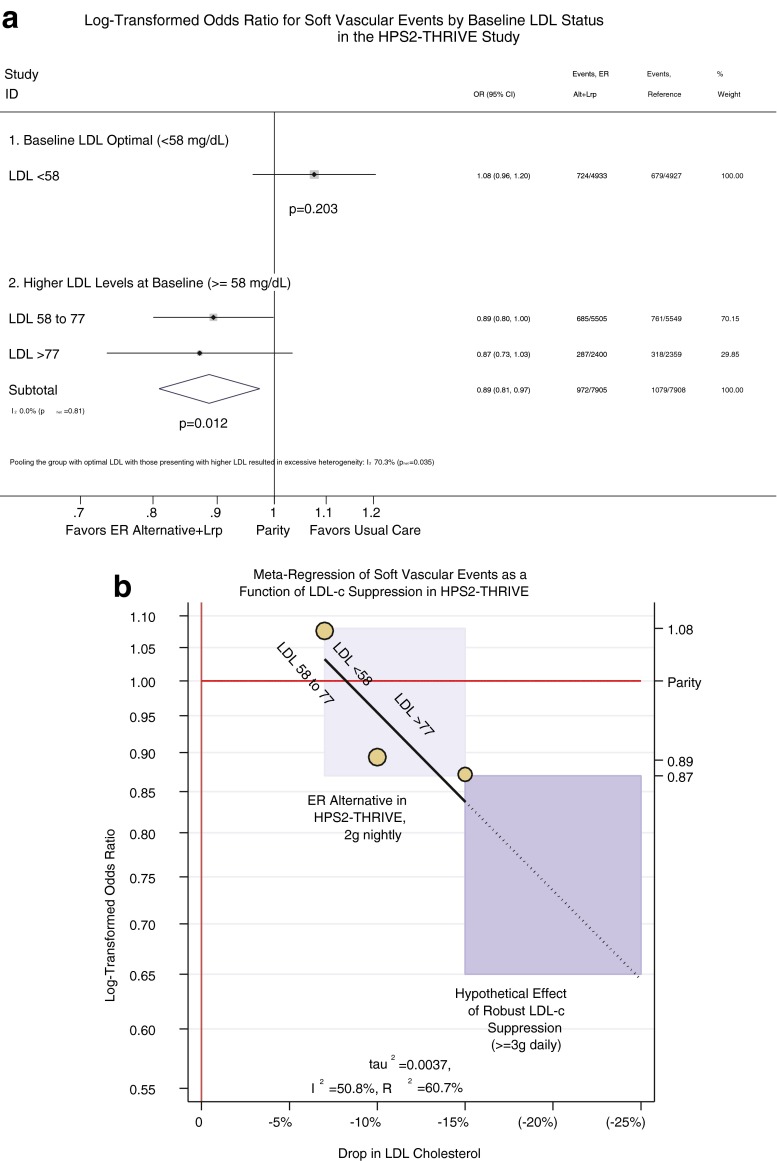
**a** Meta-analysis of odds ratio for a composite of soft vascular events from the HPS2-THRIVE study, stratified by baseline LDL-C. Importantly, pooling groups across the baseline LDL-C led to a high degree of heterogeneity (*I* ^2^ = 70.3 %, *p* < 0.05), thus arguing against pooling all three groups. One group (LDL-C < 58 mg/dL) had an OR > 1.0, and the other two (LDL-C ≥ 58 mg/dL) had OR < 1.0, and the latter two had almost identical OR’s (0.89 and 0.87). Affirming this, when we pooled the latter two groups, heterogeneity was minimized (*I* ^2^ = 0 %, *p* = 0.8). Again, those with optimized LDL-C (i.e., LDL-C < 58 mg/dL) differed from those with higher LDL-C, having no discernable benefit from ERN + laropiprant compared to placebo (OR = 1.08, *p* = 0.2). In contrast, those with LDL-C ≥ 58 mg/dL appear to benefit from ERN + laropiprant (OR = 0.89, CI = 0.81 to 0.97, *p* = 0.01). Oddly, the study was severely skewed toward people with lower LDL-C, with a minority having LDL-C > 77 mg/dL. These results suggest that a study enrolling people with higher LDL-C (e.g., LDL-C > 70 or >100 mg/dL) might be the ideal way to test the incremental benefit of the ER alternative. **b** Meta-regression between log-transformed odds ratio for soft vascular events from the HPS2-THRIVE and percent change in LDL-C based on baseline LDL-C. The findings from (**a**) suggest the study’s primary aim suffered from targeting a population who does not necessarily benefit from further LDL lowering. Conversely, the apparent benefit among those with suboptimal LDL-C suggests higher degrees of LDL suppression do confer benefits, in accordance with the LDL hypothesis. To illustrate this, we conducted a meta-regression showing fewer events with more aggressive LDL-C suppression (*R* ^2^ = 61 %). Though we used linear regression, there was heterogeneity (*I* ^2^ = 51 %), suggesting a nonlinear model may fit better. In any case, the relationship is consistent with the LDL hypothesis. As such, this promising result might be exploited to greater effect using the established cardioprotective regimen or better yet, the lipid-targeting strategy of niacin. Both strategies achieve more aggressive LDL-C lowering. According to the LDL hypothesis, this should build upon the promising event reductions from the ER alternative in HPS2-THRIVE. The dark box on the graph represents the hypothesized effect of more robust niacin regimens. Based on HPS2-THRIVE, we predict that a future trial using aggressive LDL-C suppression would have a result somewhere within the dark box (i.e., OR < <0.87)

Intuitively, niacin suppressed LDL-C much more in those with high baseline LDL-C: −7 mg/dL for LDL-C ≤ 58 mg/dL vs. −15 mg/dL for LDL-C ≥ 77 mg/dL. In accordance with the LDL hypothesis, one would expect those with more substantial LDL-C suppression fare better. As predicted, baseline LDL-C drove substantial heterogeneity in vascular outcomes, indicating some groups had more of a response than others (*p* < 0.05). Indeed, the group with baseline LDL-C < 58 mg/dL had no benefit from niacin, consistent with their optimal LDL (OR = 1.08, 95 % CI = 0.96 to 1.20, Fig. 2a). In contrast, pooling the groups with LDL ≥ 58 mg/dL eliminated heterogeneity (now *p* = 0.8), and importantly, those with LDL ≥ 58 mg/dL had fewer vascular events (OR = 0.89, 95 % CI = 0.81 to 0.97, NNT 74, *p* = 0.012). Similar benefits were seen in those with suboptimal ApoB (Supplemental Figure).

Thus, although HPS-THRIVE proved ERN + laropiprant is no “panacea,” it does point to efficacy among people who actually have suboptimal LDL-C. Since that is precisely what one would predict from the LDL hypothesis, we performed a meta-regression of event reduction as a function of LDL-C suppression (Fig. 2b). Though this supports a relationship (*R*^2^ = 61 %), there was some heterogeneity suggesting a threshold effect as an alternative to a strictly linear dose response. In either case, the results accord with the LDL hypothesis. Given unimpressive LDL-C reductions from up to 2 g of the ER alternative, this naturally raises an important question: Since the established cardioprotective regimen suppresses LDL-C more aggressively, could we achieve more robust CHD benefits with the reference dose of 3 g daily? Would a lipid-targeting strategy work even better? If the LDL hypothesis is correct, one would expect a trial pushing the dose to maximize LDL-C or non-HDL-C suppression to fare much better. Nevertheless, the HPS2-THRIVE subset reassures clinicians that in all likelihood the ER alternative would benefit the not-exactly uncommon group of patients with LDL-C ≥ 58 mg/dL or ApoB ≥ 60 mg/dL despite statin therapy.

Apart from efficacy, safety took on unusual significance for HPS2-THRIVE because of the laropiprant component. As background, the FDA approved 39 niacin drug/dose combinations. In a stunning departure, when laropiprant was proposed to be added to niacin, the FDA rejected the combination in 2008. In retrospect, this is understandable considering laropiprant was a novel chemical entity lacking long-term safety from large populations. Thus, rejection of the only niacin formulation with laropiprant motivated HPS2-THRIVE, providing the first major long-term safety profile. The trial validated safety concerns by a high prevalence of adverse effects. Some were unexpected based on niacin literature, notably, serious bleeding events (2.5 vs. 1.9 %, *p* < 0.001), including the intracranial and gastrointestinal bleeding.

Disconcertingly, laropiprant absent niacin inhibits platelet responsiveness to collagen and enhances the antiplatelet effects of aspirin and clopidogrel [48, 49]. Accordingly, changes in platelet function in silico translated to prolonged bleeding time in vivo among dyslipidemics exposed to laropiprant absent niacin. Indeed, bleeding time was underestimated because the protocol censored the maximal bleeding time, but laropiprant-exposed subjects were still bleeding when they reached the contrived maximum [48]. Hence, laropiprant’s penchant to prolong bleeding implicates the novel chemical entity in the serious bleeding events in HPS2-THRIVE. The latter also found an unexpected increase in infections (8 vs. 6.6 %, *p* < 0.001). Accordingly, by antagonizing the prostaglandin D2 (PGD2) receptor DP1, laropiprant may enhance the PGD2-mediated regulation of the immune response via a distinct PGD2 receptor-CRTH2 and increase propensity to infections [50]. Thus, pre-clinical work supports the concept that laropiprant may have promoted bleeding and infections in HPS2-THRIVE. Far from assuaging safety concerns, HPS2-THRIVE instead raised new concerns, translating theoretical safety problems into confirmed serious adverse events, seemingly validating the FDA’s denial.

Strictly speaking, the study design does not permit one to separate adverse effects from laropiprant and the ER alternative. Although IR-niacin has been studied for many decades longer than the ER alternative, extensive experience with the former does not necessarily rule out novel side effects from the latter, especially since the dosing regimen is so different (cf. Table 1). The high incidence of adverse events in HPS2-THRIVE prompted a letter attempting an unplanned post hoc analysis of the aborted AIM-HIGH trial comparing serious adverse events on the ER alternative to low-dose IR-niacin [51]. In contrast to HPS2-THRIVE, the incidence of serious bleeding events did not differ (3.4 % on the ER vs. 2.9 % on IR-niacin, *p* = 0.36). However, serious infections occurred with similar frequency as HPS2-THRIVE (8.1 on ER vs. 5.8 % on IR-niacin, *p* = 0.008). The authors cite several factors that mandate caution while interpreting the AIM-HIGH findings, concluding “lacking additional clinical and scientific confirmation, we believe that they should be considered to be provisional and exploratory.” Nevertheless, we will probably never be able to decisively attribute the serious adverse events to laropiprant alone, since it was promptly withdrawn from the global market. Other adverse effects in HPS2-THRIVE were predictable from the niacin literature, including gastrointestinal adverse effects (4.8 vs. 3.8 %, *p* < 0.001), new-onset diabetes (5.7 vs. 4.3 %, *p* < 0.001), and worsened glycemic control among diabetics (11.1 vs. 7.5 %, *p* < 0.001). Though niacin’s propensity to perturb glucose homeostasis has been widely reported, the effects are inconsistent in occurrence, persistence, and severity [52, 53]. Notably, statins consistently raise glucose, especially intensive statin therapy [54–56]. Though not novel, the HPS2-THRIVE findings of the effect on diabetes with combination therapy reaffirm longstanding advice that niacin can be used cautiously in pre-diabetics and diabetics.

### The AIM-HIGH Trial: An Aborted Test of the HDL Hypothesis

Whereas HPS2-THRIVE assessed the overall utility of the alternative as an adjunctive lipid-altering drug, AIM-HIGH tried to dissect its HDL-raising potential from its LDL-lowering properties. Doing so addresses the HDL hypothesis, namely, that raising HDL-C per se would prevent cardiovascular events. This study was motivated in part by the HDL Intervention Trial (HIT) trial, where as little as 6 % higher HDL-C from a fibrate prevented hard CHD events (RRR 22 %) absent LDL-C effects in patients whose primary lipid problem was low HDL-C [57]. Tellingly, CHD prevention was related to raising HDL-C [58]. Likewise, the Helsinki Heart Study (HHS) also found gemfibrozil cardioprotective [59]; again, CHD benefit was a function of raising HDL-C [60]. Since niacin is much more effective at raising HDL-C, it presented an obvious choice to exploit the apparent benefits of raising HDL-C. Accordingly, AIM-HIGH enrollees had cardiovascular disease and low HDL-C, but optimal LDL-C levels. Participants initially took the ER alternative 1500–2000 mg all at night, and after titration, were randomized to continue the ER alternative or switch to IR-niacin 100–150 mg/night. The decision to give the comparator group an active intervention rather than a bona fide placebo was motivated by a desire to maintain blinding by inducing NASTy symptoms in both groups. Since the purpose of the trial was to isolate the HDL-raising effect of niacin, the investigators had to account for LDL-lowering by niacin, necessitating rigorous efforts to equalize LDL-C levels between groups post-randomization. Thus, throughout the trial, LDL-C was forced below 80 mg/dL in both groups by titrating simvastatin to 80 mg/dL and adding ezetimibe 10 mg/day as needed. This was complicated by an FDA “black box” warning strongly discouraging simvastatin 80 mg/dL after the trial started.

**Fig. 3:**
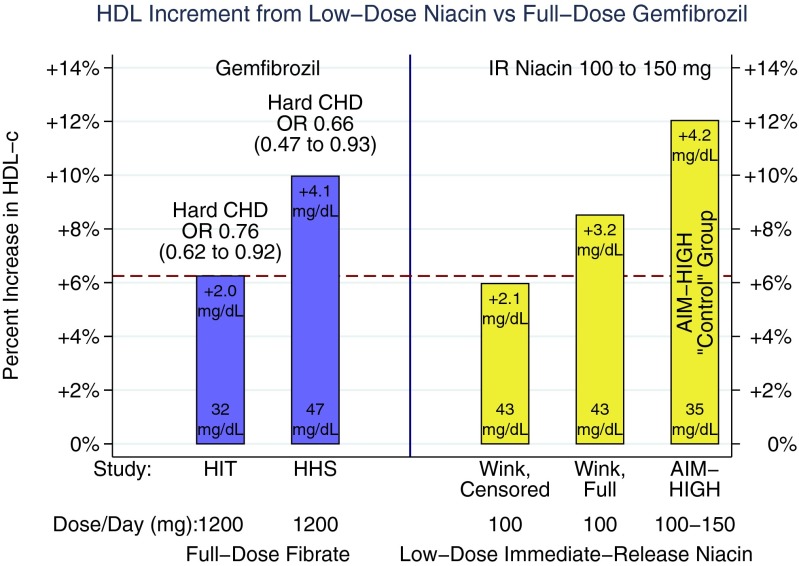
*Left panel* indicates two outcome trials affirmed the HDL hypothesis with the fibrate gemfibrozil, HIT, and HHS [58, 59]. The HIT study is more comparable to AIM-HIGH, having enrolled high-risk patients with low HDL-C at the baseline. Thus, the *dashed red line* provides a benchmark for an HDL-C increment expected to prevent CHD events. Both the HIT and HHS demonstrated the fibrate prevented hard CHD (OR = 0.66 to 0.76) with an HDL-C increment of 6 to 10 %. *Right panel* indicates two studies where low-dose niacin was added to a statin-treated background demonstrating low-dose niacin achieves similar to HDL-C increments as the fibrate studies [18••, 61]. The study by Wink et al. reported the HDL-C increment from low-dose niacin in two ways. They censored their dataset to exclude one subject on low-dose niacin who had an unusually robust rise in HDL-C, but also reported the full dataset. This suggests a variable response to low-dose niacin, where some subjects are hyper-responders and cause analytical problems due to their very high increments. The AIM-HIGH “control” group received even more immediate-release niacin than in the Wink study, in most cases 150 % of the Wink dose. Not surprisingly, the stronger doses used in AIM-HIGH stimulated a prodigious rise in HDL-C that is not only higher than the Wink study but also considerably higher than the benchmark HIT study. Remarkably, by pushing the dose, the AIM-HIGH investigators managed to *double* the increment in HDL-C: +12 % (+4.2 mg/dL) in AIM-HIGH vs. +6 % (+2 mg/dL) in HIT. If the HDL hypothesis supported by the fibrate studies also translates to niacin, one would expect the AIM-HIGH “control” group to have similar benefits as HIT or HHS, and since the HDL-C increment is so much more robust, perhaps even greater benefits. Unfortunately, this invalidates the intended control group in AIM-HIGH, because the control by necessity should represent the untreated state, specifically a group lacking an HDL-raising dose of niacin, and preferably lacking niacin altogether. *IR* immediate release, *OR* odds ratio, *Hard CHD* non-fatal myocardial infarction and/or cardiac death, *HIT* HDL intervention trial, *HHS* Helsinki heart study

To most of the investigators’ shock, both niacin formulations raised HDL-C robustly: median change +9.8 % on low-dose IR-niacin after 2 years and +25 % on 2 g of the ER alternative, a net differential of about 15 %. By 3 years on low-dose IR-niacin, HDL-C rose from 34.9 to 39.1 mg/dL (+4.2 mg/dL, mean = +12 %, median = +11.8 %). On the ER alternative, HDL-C rose from 34.5 to 44.1 mg/dL (+9.6 mg/dL, mean = +28 %, median = 25 %), for a differential of +5.4 mg/dL between the two forms of niacin favoring the group on the ER alternative. To put this into context, the motivating HIT study found a +6 % rise in HDL-C prevented hard CHD events, but here, niacin was so much more potent that even the low dose of IR niacin in the intended control group inadvertently had *double* the increment of HDL-C from HIT (+12 vs. +6 %, Fig. 3). The use of such a robustly effective HDL-raising dose of the active intervention in what was to be the *control* group had a catastrophic effect on the validity of the study’s primary aim, to prove the HDL hypothesis.

Superiority of the ER alternative over low-dose IR-niacin would have affirmed the HDL hypothesis. Regrettably, the study was unable to distinguish the two active therapies, resulting in the “nightmare” scenario of an un-interpretable null result. The problem is actually worse than stated, because contaminating the control group with a highly-efficacious dose of the experimental intervention unwittingly converted the study from a controlled experiment of drug efficacy to an uncontrolled dose–response study, bizarrely lacking the requisite drug-free arm. A dose–response study requires a drug-free control to prove efficacy given a flat dose response. Without a niacin-free arm, AIM-HIGH’s null results are uninterpretable. On the one hand, the null could mean raising HDL-C 28 % with the ER alternative is no better than raising it a robust 12 % with low-dose IR-niacin, but *both* improve upon usual care, perhaps more than HIT or HHS since the starting HDL-C increment was better, hence, proving the HDL hypothesis. On the other hand, the null could mean *neither* dose is effective, failing to affirm the HDL hypothesis at these doses. So, which is it? Frustratingly, we have no way to know. Thus, foregoing a valid control group became a “show-stopper,” rendering it hard defend AIM-HIGH as a reliable test of the HDL hypothesis. Indeed, many reviewers have expressed significant misgivings about the validity of the trial’s results [62–64].

Beyond the invalid control group are several other scenarios to explain null results (Table 2). Any null result invites scrutiny of a study’s power. Adding the experimental intervention to its own control severely narrowed the HDL-C differential between IR- and ER-niacin in AIM-HIGH. Outcome benefits from narrow group-wise differences were probably well beyond the detection limit of AIM-HIGH, powered to detect a 25 % reduction in “softer” clinical events. Complicating matters, almost 20 % of patients in both groups, had previously been treated with niacin, having discontinued 30 days before enrollment. The legacy benefits of niacin seen in the CDP several years after niacin discontinuation imply niacin is a disease-modifying drug, thus fundamentally altering CHD risk for many years. Such legacy effects would have persisted throughout the foreshortened AIM-HIGH trial, reducing overall events, hence further diminishing the study’s power to detect differences in outcomes.

**Table 2:** Possible interpretations of the AIM-HIGH null result for the HDL hypothesis

Power was undermined by design, implementation, or other flaws
• The HDL hypothesis is operative, but the robust increase in HDL in the IR niacin group narrowed the HDL differential between the groups, thereby invalidating the power calculations. Thus, the AIM-HIGH null result reflects inadequate power.
• The HDL hypothesis is operative, but the motivating studies also involved unmeasured benefits that inflated the apparent HDL effect. Absent a way to identify such effects, the elaborate machinations to isolate the HDL benefit in AIM-HIGH inadvertently nullified related benefits, undermining power.
• The HDL hypothesis is operative, but the ER alternative (1) has countervailing properties that undermine CHD benefits and/or (2) fails to recapitulate essential properties of the established cardioprotective regimen that augment CHD benefits (cf Table 1). Thus, the AIM-HIGH null result occurred because the ER alternative is markedly less effective than the established cardioprotective regimen and less effective than expectations, undermining power.
• The HDL hypothesis is operative, but the effect of niacin was diluted in both groups by enrolling patients who were already treated with niacin. This is problematic because the legacy effect of niacin in the CDP trial indicates niacin is a disease-altering drug. As such, participants who were already on niacin might have fewer events than planned, undermining power calculations.
Faulty assumptions doomed the experiment
• The HDL hypothesis is operative when HDL-C rises more than 6 % (cf HIT), but has a flat dose response beyond the unexpected 12 % increase from IR niacin in the “control” group. Thus, the AIM-HIGH null occurred because further increments in HDL-C from the ER alternative were past the point of diminished returns. This implies the 12 % increase from IR niacin, like the 6 % increase from HIT, is beneficial, but to an unknown extent because there was no niacin-free control group. In this scenario, the experiment was doomed by the demonstrably false assumption that low doses of IR niacin had no impact on HDL-C, invalidating the control group.
• The HDL hypothesis is operative, but the AIM-HIGH doses of niacin were not sufficient to raise HDL enough to detect the benefit. This could be because HIT and HHS involve a second, unmeasured benefit of fibrate, or the ER alternative is simply inferior to the established cardioprotective regimen.
The experiment was compromised by bias
• The HDL hypothesis is operative, but AIM-HIGH was undermined by introducing a third cardioprotective agent (ezetimibe) in an unbalanced manner that disproportionately benefited the “control” group. Thus, AIM-HIGH was null to the extent that the “control” group received game-changing “help,” and the study could not cope with this bias, rendering null results untrustworthy.
• The HDL hypothesis is operative, but went undetected because AIM-HIGH was aborted before one of the niacin-treated groups hit an inflection point that would separate the event curves. Aborting a trial midway not only invalidates power calculations, but far worse, can introduce bias. For example, if the analytical and especially the biological assumptions used to rationalize aborting the trial were faulty, the decision to not see the protocol through completion can become a self-fulfilling/self-defeating prophecy. Thus, under this scenario, the AIM-HIGH was null due from undermining power at best and from investigator bias at worst, rendering null results especially untrustworthy.
HDL hypothesis is actually incorrect
• The HDL hypothesis is fundamentally misguided and incorrect, and the HIT and HHS regimens were primarily cardioprotective due to an unmeasured benefit of gemfibrozil rather than raising HDL-C. If so, and niacin did not recapitulate fibrates’ unmeasured benefits, both low-dose IR and the ER alternative would not have conferred benefits despite achieving HDL increments that were multiples of that of HIT. Thus, if and only if all of the prior possibilities are immaterial, the AIM-HIGH null result would argue against the HDL hypothesis.

Additionally, there were more insidious problems. For example, there were significant group-wise differences in simvastatin dose and use of ezetimibe (21.5 % on IR vs. 9.5 % on ER niacin), mandated by on-study LDL-C matching. Complicating matters, the IMPROVE-IT trial found further reduction in cardiovascular events when ezetimibe is added to statins [65••]. Thus, AIM-HIGH allowed unbalanced use of another cardioprotective agent, introducing a bias to the “control” group, insofar as that group was more likely to receive “help” that could have disproportionately affected outcomes. Compounding these challenges, AIM-HIGH was prematurely terminated after a mean follow-up of only 3 years, ostensibly for lack of expected benefit from an early peek at the data and a non-significant increase in ischemic strokes in the ER alternative group. This is a “double-whammy” because aborting the study can decimate the power to detect meaningful differences, but worse, can introduce bias. It appears the investigators assumed event curves should exhibit a visible separation at the time they made their decision, and finding none, stopped the trial. But what if that assumption was wrong? What if the curves separate later? Disconcertingly, the CDP did not show divergent event rate curves for niacin and placebo until well after 4 years. Thus, AIM-HIGH’s decision to stop the study prematurely may have irretrievably biased results.

Of course one of the many possibilities behind a null result is that the HDL hypothesis is incorrect. Given so many unchallenged alternatives, we find the null results of AIM-HIGH uninterpretable. Barring new post hoc subanalyses that might distinguish the groups, results from AIM-HIGH should be considered hypothesis-generating at best.

### Would the ER Alternative Help When Statins Don’t Deliver?

Regrettably, neither HPS2-THRIVE nor AIM-HIGH decisively addresses the gap left in the statin-averse, non-responders, and incomplete responders, almost half the total population at risk of cardiovascular disease. Thus, we have no way to know if the exploratory regimen with the ER alternative is helpful in that population. In contrast, at 1000 mg thrice daily, we do have evidence supporting IR-niacin and pentaerythrityl tetranicotinate to prevent CHD events when statins do not deliver. Likewise, since both trials of the ER alternative studies enrolled subjects with fairly low LDL-C, neither adequately assessed whether the exploratory alternative regimen would prove cardioprotective when LDL-C is uncontrolled. That said, the HPS2-THRIVE subgroup with LDL ≥ 58 mg/dL or ApoB ≥ 60 mg/dL strongly suggests the ER alternative would prove beneficial in people with suboptimal lipids.

### The Lipid-Targeting Strategy

The other alternative to the established cardioprotective regimen has been to titrate niacin to achieve specific lipid targets. Though this has been tested in small studies of atherosclerosis, the strategy has not found the backing to be scaled up to larger outcomes trials. Thus, while atheroprotective, we cannot conclude that the lipid-targeting approach is cardioprotective. Using ≥2–3 g/day, the FATS, HATS, CLAS, and AFREGS trials affirmed decisive lipid benefits [10–13]. Using higher doses of niacin than in AIM-HIGH or HPS2-THRIVE, these studies achieved much more robust differentials in HDL-C (+25 to +40 %) accompanied by improvements in coronary atherosclerosis. The differential between the IR- and ER-niacin arms of AIM-HIGH only resulted in a 15 to 16 % differential in HDL-C, much less than the lipid-targeting studies. What if it actually takes an HDL-C differential on the order of +25 to +40 % to distinguish a benefit on cardiovascular events? If so, the decision to abandon the atheroprotective lipid-targeting strategy in favor of the ER alternative with its severe dose restriction may have doomed AIM-HIGH and HPS2-THRIVE, as they had nothing more than inconsequential niacin doses to work with. There is a difficulty in interpreting the lipid-targeting strategy, because these studies also achieved significant LDL-C changes. Hence, atheroprotection cannot be attributed to HDL-C or LDL-C changes separately. Admittedly, that distinction would be somewhat academic if the strategy proved cardioprotective.

### If Not by Raising HDL, How Does Niacin Prevent CHD Events?

The first outcomes trial of the HDL hypothesis outside the fibrate class was a wash, since the troubled AIM-HIGH study did not clearly confirm or deny a role for raising HDL-C. For the sake of discussion, suppose AIM-HIGH was a valid null that ruled out the HDL hypothesis. How could we rectify that with compelling evidence that niacin is cardioprotective? The simple answer would be that the ER alternative is so inferior as to be inert. Perhaps AIM-HIGH and HPS2-THRIVE were hamstrung by capping the ER alternative at 2 g/day, thus limiting the lipid suppression by underdosing.

A more nuanced answer returns us full circle: niacin is cardioprotective to the extent it suppresses cholesterol, as illustrated in Fig. 1b. By this model, CDP and SIHDS prevented CHD because they dosed niacin to robustly lower cholesterol, whereas HPS2-THRIVE and AIM-HIGH delivered modest cholesterol reductions owing to sparing use of niacin. Indeed, some investigations using ≤2 g/day of the ER alternative failed to show LDL-C benefits at all, even after several months [66, 67]. Especially viewed in the context of all four niacin outcome studies, both new studies nicely accord with the LDL hypothesis. First, the HPS2-THRIVE affirmed patients with optimal baseline LDL-C or ApoB had little LDL suppression and accordingly, no benefit. Strikingly, the more “clinical” population with suboptimal lipids had greater LDL-C suppression and, accordingly, benefit from the ER alternative, just as the LDL hypothesis predicts. Second, the null result from the AIM-HIGH study suggests a benefit from LDL suppression, because *both* groups underwent intensive, fastidious LDL-C management to suppress LDL-C below 80 mg/dL. One group reached that target by driving simvastatin all the way to 80 mg, whereas the other added 2 g of the ER alternative first, and both groups utilized ezetimibe to reach the target. Consider this: What does it mean that two aggressive strategies to further suppress LDL-C did just that, and then achieved identical effects on cardiovascular outcomes?

Parity of CHD events after equalizing LDL-C implies adding even a modestly LDL-lowering dose of niacin actually prevents CHD to the same extent as driving simvastatin all the way to 80 mg to force LDL-C below 80 mg/dL. Thus, “neutral” CHD outcomes from AIM-HIGH actually validate *either* method to force LDL that low. This is actually very helpful information to clinicians who might be concerned about putting a given patient on 80 mg of simvastatin for fear of harmful effects. As an eerily apt affirmation of this real-world concern, during the AIM-HIGH simvastatin 80 mg became strongly discouraged by the FDA due unacceptable risks, underscoring the frustrating reality that maximizing statins may be easier said than done for many. In conclusion, the overall evidence suggests niacin prevents CHD events by the cholesterol hypothesis after all and as such should be used to suppress the atherogenic lipoproteins rather than raise HDL-C.

### If Used At All, How Should Niacin Be Used?

If niacin is to be used to prevent MI, we find it harder to support the exploratory regimen based around the ER alternative. Instead, the overall evidence supports a return to the established cardioprotective regimen, namely 1 g thrice daily with meals. Practically, we find it surprisingly easy to switch patients from the ER alternative 2 g nightly to IR-niacin 1 g thrice daily, probably because they have substantial tolerance to NASTy symptoms by the time they accommodate 2 g of the ER alternative. For patients intolerant, averse, or non-responsive to statins, this regimen remains evidence-based monotherapy to prevent MI, whereas under-dosing the ER alternative before the overnight fast provides little such assurance. Whether niacin per se would benefit statin-responsive patients remains unanswered, since the established cardioprotective regimen has never been tested against a statin background. For that matter, when introduced the statins were never subjected to the same standard of being formally tested against a niacin background to determine incremental benefit. Thus, formal testing of incremental effects of new lipid drugs remains in its infancy, with much work to be done.

We submit the exploratory ER regimen (≤2 g/day) has thus far failed to impress because it strays so far from the established regimen, but not because of the delayed-release formulation itself. Since the niacin pro-drug pentaerythrityl tetranicotinate also delays niacin release, niacin release rates may well prove immaterial. Accordingly, were the established cardioprotective regimen tested against a statin background, we predict even the ER alternative dosed 1 g thrice daily with meals would prevent CHD among subjects with suboptimal lipids, much as the longer-acting pentaerythrityl tetranicotinate did. That said, we suspect a more efficacious approach would be to combine the established cardioprotective regimen with the lipid-targeting strategy. Thus, subjects randomized to niacin would receive the established cardioprotective regimen as a condition of enrollment, but would then titrate upwards if they were short of study-determined non-HDL-C and HDL-C goals, capped at the highest tolerated approved dose (i.e., up to 2 g thrice daily).

## Conclusions: Whither Niacin?

The spectacular failure of the exploratory ER alternative introduces a new barrier to progress. It is tempting to re-appropriate failure of the ER alternative into a more general failure of the established cardioprotective regimen, notwithstanding compelling evidence to the contrary (cf. Fig. 1). As an analogy, we could appreciate how frustration over the inopportune failure of a $20 knockoff Rolex could damage the Rolex brand, unless the buyer were sufficiently streetwise to distinguish the knockoff from the genuine article. We fear further research might be chilled unless a broad coalition of stakeholders were open to the possibility that the profoundly disappointing results from the ER alternative in AIM-HIGH and HPS2-THRIVE do not generalize to the duly-established cardioprotective regimen. Failing that, developers and their funders who are already banking on that prospect could exploit the intense “buyer’s remorse” for the ER alternative and thereby blaze a new trail by proving the merits of novel niacin mimetics with little competition from niacin itself. Stoking an ongoing failure to distinguish the dubious exploratory alternative from the established cardioprotective regimen could quash competition from niacin in perpetuity. Thus, by delving deeper into the overall evidence and skirting prior pitfalls, canny prospectors could effectively open a goldmine at the ER alternative’s grave site. In part II of this review, we will walk through how several developers have leveraged the overall pre-clinical and clinical evidence to develop novel niacin mimetics, concluding with a review of several mimetics that have already demonstrated encouraging lipid effects in small, early phase human trials.

## Electronic supplementary material

ESM 1 **A**: Meta-analysis of odds ratio for a composite of soft vascular events from the HPS2-THRIVE study, stratified by baseline ApoB. Importantly, pooling groups across the baseline ApoB led to a high degree of heterogeneity (I2 62%%, p=0.07). Since the test for heterogeneity has low power, a p value <0.1 is a reasonable indicator of significant heterogeneity. Thus, the I2 argues against pooling all three groups. One group (ApoB<60mg/dL) had an OR > 1.0, and the other two (ApoB>60mg/dL) had OR < 1.0, and the latter two had similar OR’s (0.93 and 0.89). Affirming this, when we pooled the latter two groups, heterogeneity was minimized (I2 0%, p=0.6). Again, those with optimized ApoB (i.e. ApoB <60 mg/dL) differed from those with higher ApoB, having no discernable benefit from ER niacin+laropiprant compared to placebo (OR 1.09, p=0.2). In contrast, those with ApoB>60 mg/dL appear to benefit from ER niacin+laropiprant (OR 0.91, CI 0.84 to 0.99, p=0.03). A study enrolling people with higher ApoB (e.g. ApoB > 60 or > 70 mg/dL) might be the ideal way to test the incremental benefit of the ER alternative. **B**: Meta-regression between log-transformed odds ratio for soft vascular events from the HPS2-THRIVE and percent change in LDL-C based on baseline ApoB. The findings from Panel A suggest the study’s primary aim suffered from targeting a population who does not necessarily benefit from further LDL lowering. Conversely, the apparent benefit among those with suboptimal ApoB suggests higher degrees of LDL suppression do confer benefits, in accordance with the LDL hypothesis. To illustrate this, we conducted a meta-regression showing fewer events with more aggressive LDL-C suppression (R2 89%). There was little heterogeneity (I2 15%), supporting a linear fit. Importantly, the relationship is consistent with the LDL hypothesis. As such, this promising result might be exploited to greater effect using the established cardioprotective regimen or better yet, the lipid-targeting strategy of niacin. Both strategies achieve more aggressive LDL-C lowering. According to the LDL hypothesis, this should build upon the promising event reductions from the ER alternative in HPS2-THRIVE. The dark box on the graph represents the hypothesized effect of more robust niacin regimens. Based on HPS2-THRIVE, we predict a future trial using aggressive LDL-C suppression would have a result somewhere within the dark box (i.e. OR<<0.89). (JPEG 1.81 mb)

High resolution image file (EPS 908 kb)
