# Supplementary figures and images for: Niacin Alternatives for Dyslipidemia: Fool’s Gold or Gold Mine? Part I: Alternative Niacin Regimens
Source: Curr Atheroscler Rep. 2016 Feb 15;18:11. doi: 10.1007/s11883-016-0563-8 (PMC4753247; doi:10.1007/s11883-016-0563-8)

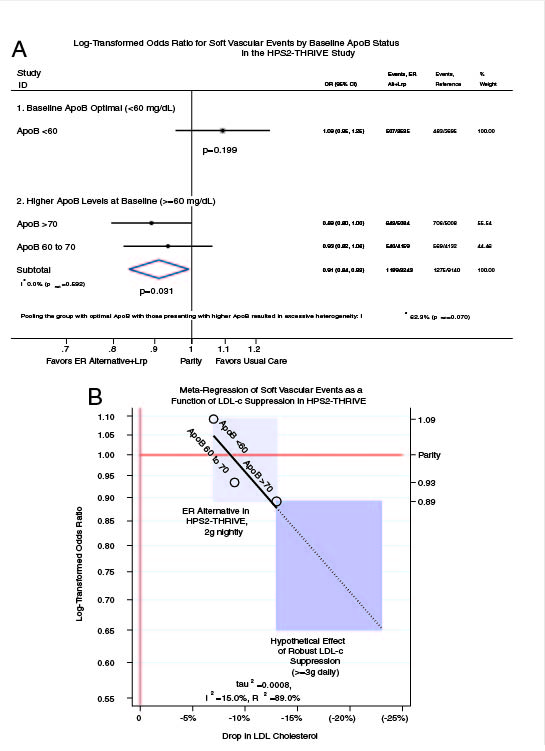

Supplement: Supplementary file 1 — A: Meta-analysis of odds ratio for a composite of soft vascular events from the HPS2-THRIVE study, stratified by baseline ApoB. Importantly, pooling groups across the baseline ApoB led to a high degree of heterogeneity (I2 62%%, p=0.07). Since the test for heterogeneity has low power, a p value <0.1 is a reasonable indicator of significant heterogeneity. Thus, the I2 argues against pooling all three groups. One group (ApoB<60mg/dL) had an OR > 1.0, and the other two (ApoB>60mg/dL) had OR < 1.0, and the latter two had similar OR’s (0.93 and 0.89). Affirming this, when we pooled the latter two groups, heterogeneity was minimized (I2 0%, p=0.6). Again, those with optimized ApoB (i.e. ApoB <60 mg/dL) differed from those with higher ApoB, having no discernable benefit from ER niacin+laropiprant compared to placebo (OR 1.09, p=0.2). In contrast, those with ApoB>60 mg/dL appear to benefit from ER niacin+laropiprant (OR 0.91, CI 0.84 to 0.99, p=0.03). A study enrolling people with higher ApoB (e.g. ApoB > 60 or > 70 mg/dL) might be the ideal way to test the incremental benefit of the ER alternative. B: Meta-regression between log-transformed odds ratio for soft vascular events from the HPS2-THRIVE and percent change in LDL-C based on baseline ApoB. The findings from Panel A suggest the study’s primary aim suffered from targeting a population who does not necessarily benefit from further LDL lowering. Conversely, the apparent benefit among those with suboptimal ApoB suggests higher degrees of LDL suppression do confer benefits, in accordance with the LDL hypothesis. To illustrate this, we conducted a meta-regression showing fewer events with more aggressive LDL-C suppression (R2 89%). There was little heterogeneity (I2 15%), supporting a linear fit. Importantly, the relationship is consistent with the LDL hypothesis. As such, this promising result might be exploited to greater effect using the established cardioprotective regimen or better yet, the lipid-targeting strategy o [file 11883_2016_563_Fig1_ESM.jpg]
